# Molecular dynamics study on the effect of the N1 neuraminidase double mutant G147R/H274Y on oseltamivir sensitivity†

**DOI:** 10.1039/d4ra07713j

**Published:** 2024-12-10

**Authors:** Ardiana Ilham Nurrohman, Hery Suwito, Ni Nyoman Tri Puspaningsih, Kautsar Ul Haq

**Affiliations:** a Bioinformatics Research Group, University-CoE-Research Center for Bio-Molecule Engineering (BIOME), Universitas Airlangga Surabaya 60115 Indonesia; b Proteomic Laboratory, University-CoE-Research Center for Bio-Molecule Engineering (BIOME), Universitas Airlangga Surabaya 60115 Indonesia; c Department of Chemistry, Faculty of Science and Technology, Universitas Airlangga Surabaya 60115 Indonesia kautsar.ul.haq@fst.unair.ac.id

## Abstract

Inhibition of neuraminidase is the most prominent target in influenza medication using oseltamivir as an inhibitor. However, the emerging resistance of neuraminidase toward oseltamivir due to mutation reduces the efficacy of oseltamivir. The generally reported mutation is a single mutation at H274Y, which declines the sensitivity of oseltamivir by almost 900 folds compared to the wild-type variant. Moreover, an additional mutation at G147R increases the resistance by more than 2000 folds. However, sufficient studies on the resistance mechanism of this variant have not yet been reported. Therefore, we simulated four neuraminidase proteins comprising wild-type (WT), G147R, H274Y, and G147R/H274Y using molecular dynamics simulation to disclose the binding mechanism of oseltamivir. Trajectory analysis was conducted to reveal structural stability and flexibility. Furthermore, end-point free binding energy calculations were conducted. The energy decomposition of each residue was also calculated. The end-point energy calculation showed a similar result to that of experimental data. The energy decomposition analysis revealed that G147R/H274Y showed significant reduction in oseltamivir (OST) interaction with R118. Salt-bridge disruption caused by R224–E276 was also observed. Modification to enhance the polarity of the inhibitor might be useful in overcoming these changes. However, it should be noted that such changes could worsen the pharmacokinetic property of the inhibitor. It is hoped that these findings will provide useful insights for the development of an anti-influenza drug that can withstand the mutant variant.

## Abstract 2

Mutation affecting oseltamivir sensitivity was studied using molecular dynamics simulation to disclose the resistance mechanism.

## Graphical abstract

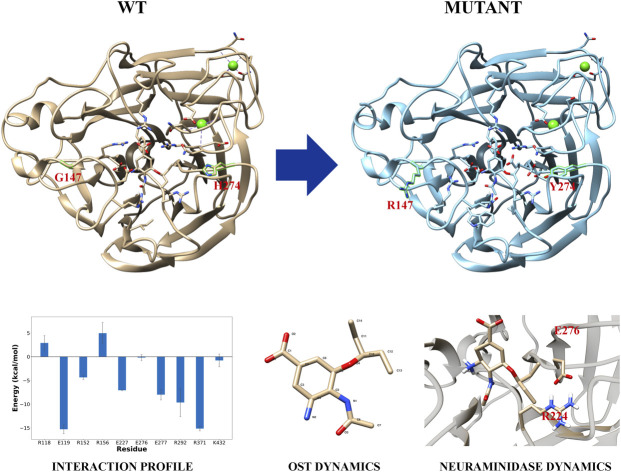

## Introduction

Influenza remains a global threat and seasonal epidemic in various parts of the world. Since its emergence, influenza has claimed millions of human lives.^1–4^ Neuraminidase is the most prominent target in influenza treatment. Neuraminidase plays a role in releasing new virions from host cells by cleaving sialic acid on the cell membrane.^1,5,6^ Inhibition of this enzyme disrupts the spread of the virus to new hosts, thereby hindering its development.^1,2,6^

Oseltamivir (OST) is the most widely used drug targeting neuraminidase for the treatment and prevention of influenza owing to its ease of oral administration.^3,6,7^ However, extensive use of the drug has raised the risk of resistance in influenza. In the decade following OST administration, symptoms of resistance to OST began to emerge. By mid-2008, 15% of circulating H1N1 virus isolates were resistant to OST globally. Later, in the winter of 2008–2009, more than 90% of H1N1 isolates were resistant to OST. Data from the Centers for Disease Control (CDC, USA) indicated that by April 2009, before the 2009 pandemic outbreak, more than 99% of virus isolates were resistant to OST. Fortunately, during the H1N1 2009 pandemic, which spread from April 2009 to January 2010, nearly all H1N1 virus variants could be treated with OST. The disappearance of resistant mutant variants during the 2009 pandemic was thought to be due to them being replaced by OST-sensitive H1N1 subtype variants.^8^ Nevertheless, the risk of the emergence of resistant variants from the H1N1 subtype after the 2009 pandemic remains a concern that needs to be anticipated.

Several mutations in 2009 pandemic H1N1 neuraminidase have been reported with varying reductions in its sensitivity toward OST. Point mutations at E119D,^9–11^ R152K,^9,11^ D199E/Y,^11–13^ I222K/R,^12,14–18^ R292K,^9,19^ I427T,^13^ and S246R^20^ cause a significant decrease in OST inhibition, ranging from 13 to 90 folds (All mutations in N2 numbering unless stated otherwise). In contrast, point mutations at N294S,^9,21,22^ P459T,^11^ and H274Y^23–32^ cause more severe reductions in OST inhibition, up to more than 200 folds. The H274Y mutation is the most commonly encountered resistant variant, resulting in a reduction in oseltamivir sensitivity by more than 900 folds.^8,33^ The H274Y mutation is intriguing because it does not occur at the active site of neuraminidase but instead near the active site residues, yet it causes a severe reduction in oseltamivir sensitivity.

Additionally, double-point mutations in combination with the H274Y mutation have also been reported to cause decreased oseltamivir sensitivity, including H274Y + I436N,^34^ D199N + H274Y,^30,35^ E119A/D/G + H274Y,^9,10,19^ I222K/R/V + H274Y,^14,18,21,26,36^ S246N + H274Y,^37^ and G147R + H274Y.^20^ The addition of a single-point mutation in the H274Y mutant variant generally results in a more severe decrease in oseltamivir sensitivity, up to more than 10 000 folds. Interestingly, the single-point mutation at G147R does not show a significant change in oseltamivir sensitivity.^33,38^ However, the addition of the G147R mutation in the H274Y mutant variant causes a further reduction in oseltamivir sensitivity of up to more than 2000 folds.^33^ This mutation was first detected in an immunocompromised patient in Japan. Furthermore, the single-point mutation at G147R is reported to have receptor-binding activity typically seen in hemagglutinin. This dual function allows the virus to enter cells even if its hemagglutinin system is disrupted. However, this variant remains susceptible to OST inhibition.^38^ The combination of this activity with OST resistance from the H274Y mutation poses a formidable risk for the spread of OST-resistant influenza infections. Unfortunately, research on this variant is still limited.

The structure of the double mutant neuraminidase G147R + H274Y has not yet been crystallized. This may be the reason why research on this mutant is still lacking. Nonetheless, structural modelling can be used as an alternative approach to modelling the mutant structure. Some early studies on the structural modelling of neuraminidase during the 2009 H1N1 pandemic also showed promising results in modelling the structure and were used for screening potential drug candidates.^39,40^ Additionally, molecular dynamics simulations can be combined to reveal molecular phenomena occurring in protein–ligand interactions that have been modelled.^41,42^ Molecular dynamics simulations offer the advantage of revealing dynamic molecular interactions in protein structures that cannot be explained by static crystal structures.^43,44^

Molecular dynamics simulation studies have revealed that larger residue replacement at position 274 disrupts the salt bridge formed by E276 and R224, which accommodates the pentoxyl moiety in OST.^42^ Another study on the binding free energy in the double mutation I222K/H274Y also aligned with the experimental results, revealing that the binding affinity of oseltamivir to the mutant variant is lower than that of the wild-type one.^41^ Park and Jo demonstrated that the H274Y mutation increases flexibility in the pentoxyl moiety. This increased flexibility allows water infiltration into the active site, thereby weakening OST interactions with neuraminidase.^44^

In this study, the mutant structure containing G147R was modelled using the crystal structure template of the wild-type and H274Y, which are available in the protein database with PDB codes 3TI6 and 5NWE.^1,45^ The structures were simulated using the AMBER22 software package for 150 ns.^46^ Trajectory analysis of the simulation results was conducted to determine the stability and flexibility of the simulated structure. End-point energy and energy decomposition analyses were performed to study the binding characteristics of each variant. Our results aligned with previous findings showing the disruption of the salt-bridge formed by R224–E276, which accommodates the pentoxyl moiety of OST. This was also exacerbated by the decreased interaction of OST with R118, which plays a pivotal role in OST binding.

## Experimental section

### Initial structures preparation

The protein structures of the pdm2009 wild-type (WT) and H274Y mutant were obtained from the Protein Data Bank with PDB codes 3TI6 and 5NWE, respectively.^1,45^ Mutations at G147R were introduced into both structures using the Dunbrack 2010 backbone-dependent library,^47^ resulting in four structures: WT, G147R, H274Y, and G147R/H274Y. Cysteine residues forming disulfide bonds were renamed using the CYX notation suitable for AMBER22. All four protein structures were protonated at pH 7.4 using the H++ webserver,^48^ retaining their calcium ions, as the presence of calcium ions has been reported to influence the calculation of the binding free energy.^49^ The protein structure was prepared using *tLEaP* module with the FF19SB forcefield, while the structure of oseltamivir was geometrically optimized using the DFT method at the M06-2X level of theory and the 6-31G(d,p) basis set.^50^ Subsequently, OST's charges were added based on the electrostatic potential fitting technique known as RESP-fitting and parameterized using the *parmchk* module with the GAFF2 atom type.^51–53^ Each structure was neutralized using counterions. The four structures were solvated in a truncated octahedron box with the OPC solvent type, parameterized ions using appropriate parameters,^54,55^ and a box distance from the protein surface of 10.0 Å.

Each system was minimized to eliminate bad contacts using 500 steps of the steepest descent followed by 500 steps of conjugate gradient minimization, with a restraint of 100.0 kcal mol^−1^ Å^−2^ on all atoms except hydrogen, heavy atoms, and the protein backbone, except for the active site residues. During the restraint of the backbone and active site residues, the restraints were gradually released from 100.0, 50.0, 10.0, 5.0, to 1.0 kcal mol^−1^ Å^−2^, and finally, there was no restraint on any of the atoms.

### MD simulation setup

The systems were heated to 310.0 K with a collision coefficient of 1.0 ps^−1^ (ref. 56) and a restraint of 10.0 kcal mol^−1^ Å^−2^ on all protein residues and oseltamivir. The heating was performed incrementally from 0.0 K to 62.0 K, 124.0 K, 186.0 K, 248.0 K, and finally 310.0 K in a canonical ensemble system (NVT). The systems were then equilibrated in an isothermal-isobaric ensemble (NPT) at a temperature of 310.0 K, target pressure of 1.0 atm, collision coefficient of 1.0 ps^−1^, and pressure relaxation time of 2.0 ps. Hydrogen atoms were restrained with gradually decreasing restraint values from 10.0, 5.0, 1.0, 0.5, to 0.1 kcal mol^−1^ Å^−2^ every 20 ps. The restraints were completely removed in the final stage, and all the atoms were relaxed for 50 ps.

All the bonds involving hydrogen atoms were constrained using the SHAKE algorithm. Electrostatic interactions were calculated using the particle mesh Ewald (PME) method with a non-bonded interaction cut-off of 10.0 Å. The equilibrated systems underwent molecular dynamics simulation using the *pmemd.cuda* program in the AMBER22 package^46^ for 100 ns in 10 stages, with a temperature of 310.0 K, target pressure of 1.0 atm, collision coefficient of 5.0 ps^−1^, and pressure relaxation time of 2.0 ps in the periodic boundary condition (PBC) simulations. A total of three replications was performed with the same condition.

### Binding free energy calculations

Binding free energy calculations were performed using two methods: Molecular Mechanics Generalized Born Surface Area (MM/GBSA) and Molecular Mechanics Poisson–Boltzmann Surface Area (MM/PBSA), considering the stability and flexibility of each system. The MMGBSA calculations estimated the electrostatic desolvation energy using the GB^HCT^, GB^OBC1^, and GB^OBC2^ models.^57,58^ The non-polar desolvation contribution was calculated using the solvent accessible surface area (SASA) technique, Δ*G*_SA_ = 0.0072 × ΔSASA. Meanwhile, the MMPBSA calculations used an interior dielectric constant of 4.0 (ref. 59) and an exterior dielectric constant of 80.0. The non-polar desolvation contribution was calculated using the solvent accessible surface area technique with a probe radius of 1.4 Å and ΔGSA = 0.0072 × ΔSASA.^60^ Each method's correlation with the experimentally derived binding free energy values was tested. The binding free energy values were obtained from the IC_50_ values reported by Takashita *et al.*, transformed using the Cheng–Prusoff equation (eqn (1)).^33,61,62^1
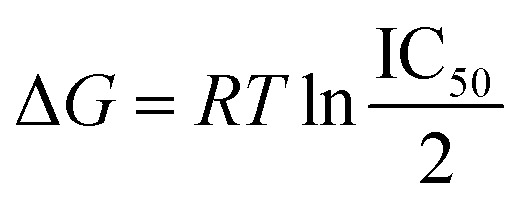
where *R* is the gas constant, *T* is the temperature, and IC_50_ is the OST inhibition concentration.^60,61^ This equation was used to calculate the difference in binding free energy with the following equation (eqn (2)):

2 ΔΔ*G*_bind_ = Δ*G*^M^_bind_ − Δ*G*^WT^_bind_

where Δ*G*^M^_bind_ is the binding free energy of the mutant and Δ*G*^WT^_bind_ is binding free energy of the wild-type. Substituting the Cheng–Prusoff equation into this equation gives eqn (3):3
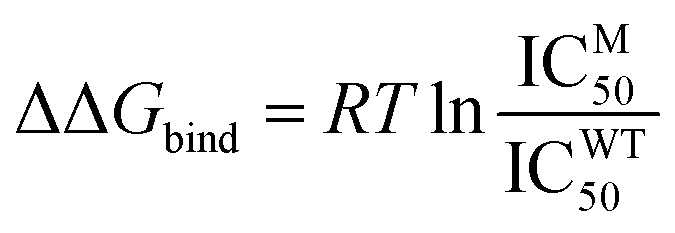
where IC^M^_50_ is the IC_50_ value of the mutant and IC^WT^_50_ is the IC_50_ value of the wild-type.

Energy decomposition calculations were performed using the results from the method that showed the highest correlation with the experimental values among the methods mentioned above.

## Results and discussions

Influenza virus, especially IAV, is still raising concern due to its threat to public health. Its easily mutated ability gives it an advantage in escaping the host immune detection and furthering its infection. G147R/H274Y is one of the mutations that occurs in the neuraminidase of IAV which further decreases the sensitivity of OST prior to H274Y mutation. The G147R mutant itself was reported to have the ability to facilitate the entry of the virus despite its hemagglutinin system being disrupted. The combination of the reduced sensitivity of OST and the latter's ability raises significant concerns about the need to address this mutant.

Here we modelled the G147R/H274Y mutant structure with the Dunbrack backbone-dependent library using Modeller software that can be integrated with Chimera.^47,63^ We used the 3TI6 and 5NWE protein structures as the G147R and G147R/H274Y template structures, respectively, because of their high resolution and good crystal quality. These modelled structures were then subjected to rigorous analysis.

### System stability and flexibility

The modelled protein needed to be evaluated to assess whether it properly represented the target protein. The system stability can be used to evaluate the quality of modelled proteins. There are many properties that can be used to evaluate system stability, with the root mean square deviation (RMSD) the most widely used to determine stability. A low value of RMSD indicates that the modelled protein structure did not undergo significant conformational changes. However, some fluctuation was still expected due to the dynamicity of protein.

**Fig. 1:**
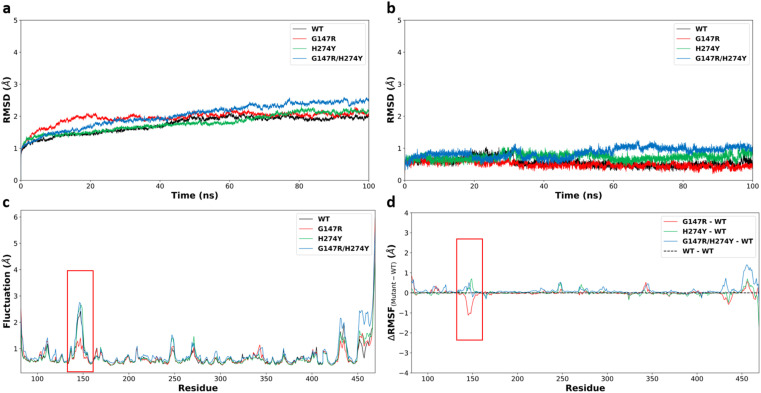
(a) Protein backbone RMSD of the four complex structures. (b) Ligand (OST) RMSD of the four complex structures. (c) Protein backbone RMSF and (d) ΔRMSF of the four complex structures, with the red box indicating the residues forming the 150-loop.

Overall, the four neuraminidase–oseltamivir complex structures remained relatively stable, with an average root mean square deviation (RMSD) of the protein below 2.0 Å, with the exception for G147R/H274Y. The four structures were relatively stable after a few nanoseconds, as indicated by the mild slope of the curve. The average RMSD values for the WT, G147R, H274Y, and G147R/H274Y structures were 1.74, 1.95, 1.78, and 2.02 Å, respectively (Fig. 1a).

The protein RMSD values primarily indicated the overall stability of the protein structure. The terminal residues of proteins, which are often flexible and not stabilized by secondary structures, tend to contribute significantly to the protein RMSD due to their inherent flexibility. Therefore, to assess the stability of oseltamivir (OST) within the active site, the RMSD values of OST were also calculated. All four structures demonstrated relatively stable OST positions within the active site, with OST RMSD values remaining below 2.00 Å (Fig. 1b). H274Y and G147R/H274Y showed some fluctuation in the OST RMSD but the overall RMSD was still below 2.00 Å. It is important to note that the RMSD stability does not always correlate directly with the binding free energy stability but can serve as an initial indicator of the changes in interactions.^42^

To identify flexible or highly mobile residues, root-mean-square-fluctuation (RMSF) calculations were performed. RMSF measures the positional fluctuations of each residue's atoms over the simulation period. The RMSF values for the backbone atoms of the WT, G147R, H274Y, and G147R/H274Y variants were relatively similar, with significant fluctuations observed around the 150-loop region. In particular, H274Y and G147R/H274Y exhibited higher fluctuations in this region, while G147R exhibited lower fluctuation compared to WT (Fig. 1c). The fluctuations in the 150-loop were expected in the neuraminidase protein structures, as this loop regulates the opening and closing of the protein's active site cavity. The 150-loop also contains the key oseltamivir binding residue D151, which interacts with the amine group of oseltamivir.^41^

Intriguingly, the mutation at position 147 appeared to make the 150-loop structure more rigid, as indicated by the lower value of RMSF of G147R than that of WT. However, H274Y exhibited a more flexible structure at 150-loop. G147R/H274Y showed a similar phenomenon to H274Y with a slightly increased flexibility.

The RMSF values also indicated high fluctuations at the termini of the protein chains. This is common due to the inherent flexibility of protein ends, which typically lack a secondary structure and are not stabilized by sufficient interactions. Such flexible residue fluctuations can contribute to high RMSD values, making the overall high RMSD values from these fluctuations negligible.

### Binding free energies

The binding free energies of the four structures were calculated using two methods: MM/GBSA and MM/PBSA. In the MM/GBSA method, three GB models were used: HCT, OBC1, and OBC2. Each method was tested for correlation against experimental values every 10 ns by calculating the Pearson coefficient of correlation. The method with the best correlation value was used for the next energy decomposition calculations.

**Fig. 2:**
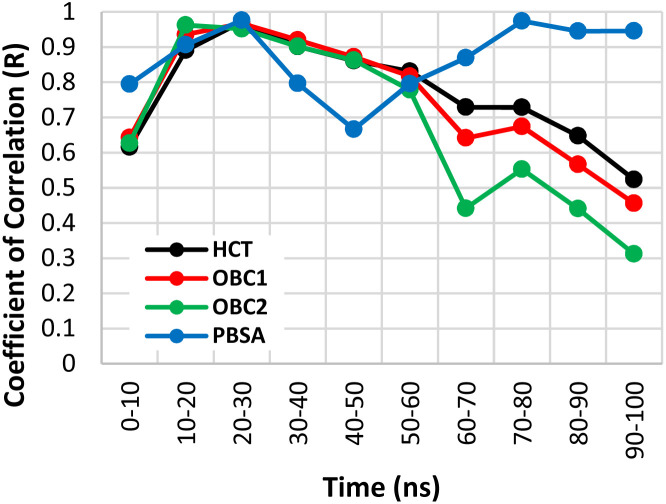
Coefficient of determination (*R*^2^) values for the free binding energy calculation methods over the simulation time.

Fig. 2 shows the Pearson coefficient of correlation of the binding free energies calculated using MM/GBSA and MM/PBSA compared to the experimental results over the course of the simulation. The coefficient of correlation was calculated by transforming the IC_50_ values obtained from Takashita *et al.* (2016)^33^ and Nguyen *et al.*^64^ using eqn (3) and the calculated Δ*G*_bind_ using eqn (2).

Generally, the coefficient of correlation of the MM/GBSA methods decreased after 30 ns simulation. The highest coefficient of correlation of MM/GBSA was obtained using GB^HCT^ with a value of 0.986 at the 20–30 ns segment. The MM/PBSA method also encountered a similar pattern with a decreased coefficient of correlation after 30 ns simulation time. However, unlike MM/GBSA, it improved again after 50 ns simulation time reaching another peak at 70–80 ns before then declining again. The first peak (20–30 ns) had a coefficient of correlation of 0.988, while the second peak (70–80 ns) had a value of 0.987. The detailed values of each variant (20–30 ns) are displayed in Table 1. Therefore, the MM/PBSA method was chosen for the energy decomposition calculations to determine the interaction profiles of the four structures: WT, G147R, H274Y, and G147R/H274Y at the 20–30 ns segment.

**Table 1:** Free binding energy of each variant calculated using MM/PBSA^a^

Variant	ΔΔ*G*_Exp_ (kcal mol^−1^)	Δ*G*_Calc_ (kcal mol^−1^)	ΔΔ*G*_Calc_ (kcal mol^−1^)
WT	0.00	−34.18 ± 0.80	0.00
G147R	0.88	−33.82 ± 1.97	0.37
H274Y	4.20	−32.27 ± 0.58	1.91
G147R/H274Y	4.84	−32.37 ± 0.44	1.82
***R* (*R*** ^**2**^ **)**			**0.99 (0.98)**

a ΔΔ*G*_Exp_ = experimental binding free energy difference to the wild-type, Δ*G*_Calc_ = calculated binding free energy, and ΔΔ*G*_Calc_ = calculated binding free energy difference to the wild-type. The calculated value is presented as mean ± standard error (*x̄* ± se).

### Energy decomposition analysis

Energy decomposition calculations were performed using the MM/PBSA method at the 20–30 ns segment, as it provided the highest correlation coefficient. Consequently, the interaction profile analysis conducted closely reflected the actual state. A threshold of ±1 kcal was used to filter residues that significantly contribute to the neuraminidase–OST interaction. An increase in energy indicated a weakening interaction of that residue with OST, whereas a decrease in energy indicated a strengthening interaction of that residue.

The interaction profile of each variant was quite distinguished, with some residues exhibiting similar patterns to one another. In this study, we only focused on the interaction change of the G147R/H274Y mutant. The most drastic change observed in the G147R/H274Y mutant was its interaction with R118. Even though the other mutants also showed reduced favourable interactions, G147R/H274Y suffered greatly, with the interaction even becoming unfavourable. A similar pattern was also observed in E227 and E276, which showed decreased favourable interactions in the mutant, yet G14R/H274Y suffered the worst. We believed that these interactions resulted from the combination effect of both mutations, which worsened the binding affinity of G147R/H274Y.

G147R/H274Y also showed a distinct pattern similar to that of G147R only at E119, R152, and K432. Regarding the interaction with E119 and R152, both G147R and G147R/H274Y, showed increased favourable interaction compared to the WT, while H274Y showed relatively the same or comparable interaction, but the interaction with K432 was reduced. Nonetheless, the sum of these interactions was still favourable for G147R and G147R/H274Y, indicating that the mutation did not always reduce the affinity, but also increased the affinity at some residue because of the interaction changes.

Furthermore, G147R/H274Y showed a similar interaction with H274Y too at R371. Both of them had slightly higher energy than that of the WT, indicating that the interaction with this residue was slightly weakened. In contrast, G147R showed a great decrease in its energy, indicating that the interaction with R371 was strongly favourable.

The most interesting changes observed were the peculiar interactions of G147R/H274Y at R156, E277, and R292 that did not match both single mutations. In R156 and E277, both G147R and H274Y showed increased energy compared to the WT, indicating the less favourable interaction with the residues. Meanwhile, G147R/H274Y had comparable energy to that of the WT, indicating that there was not much interaction change within these residues. On the other hand, the interaction with R292 was a noncomplementary combination of G147R and H274Y, where the mutation at 147 strengthened the interaction that was weakened due to mutation at 274, or *vice versa*. All of these changes in interaction with G147R/H274Y supposedly contributed to its reduced binding affinity.

Visual investigations were also conducted to observe any visible changes. The four structures were clustered at the 20–30 ns segment into 4 clusters based on their similarities using *k*-means clustering algorithm. The largest cluster was chosen as the representative model, assuming it contributed most significantly to the binding free energy calculations.

Over the four clusters obtained, there were two big clusters of each variant with their occupancy exceeding 30%. All of the variant formed two OST conformations, except G147R, which only had one conformation (Fig. 4). One conformation of OST in the WT interacted closely with the key residue of R371, which is essential in sialic acid binding. The other conformation was a bit loose towards this interaction but still maintained the interaction with the arginine triad (R118, R371, and R292), which facilitated the carboxyl moiety of the sialic acid.^49^

**Fig. 3:**
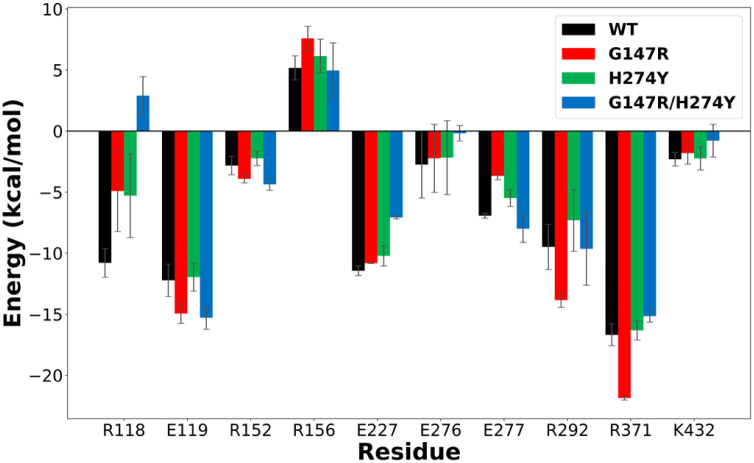
Residues that play a significant role in OST binding. The error bars indicate the standard error means of the replicates.

**Fig. 4:**
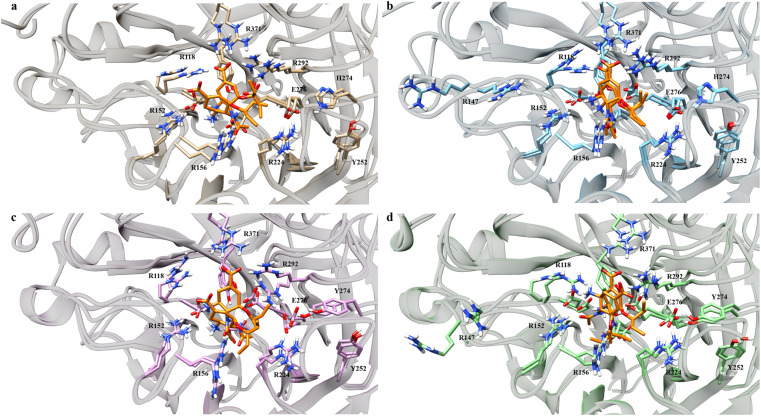
Binding poses of OST at the active site of neuraminidase. Light brown indicates the WT (a), blue indicates G147R (b), pink indicates H274Y (c) and green indicates G147R/H274Y (d). OST is depicted in orange.

On the other hand, G147R closely maintained these arginine triad interactions across all clusters, reflected by its strong interactions with R371 and R292 (Fig. 3). H274Y looked similar to that of the WT; however, the interaction profile was not quite the same. Further investigation was needed to disclose whether it underwent a similar condition as the WT. The salt-bridge interaction of R224–E276 was disrupted by steric hindrance caused by the bulkier side chain of H274Y (Fig. 4c).

In contrast, G147R/H274Y had its carboxyl moiety pointed at R292 (Fig. 4d). This change resulted in unfavourable interaction between the amino moiety of OST and the R118 residue due to electrostatic repulsion. Moreover, the pentoxyl moiety of OST drifted away from its initial region to a highly polar region. This might affect the stability of the pentoxyl moiety due to a hydrophilicity difference. Similar to H274Y, the salt-bridge interaction was also disrupted by H274Y.

### Dynamic features of the neuraminidase–OST complex

Neuraminidase is an enzyme with a highly, or at least predominantly, polar active site. This is due to the abundance of polar residues around its active site, such as arginine and glutamic acid. This characteristic was also observed in the energy decomposition of the four complex structures. We found a phenomenon similar to that described by Li *et al.* (2012),^42^ where the polar contributions correlated well with the binding free energy (see Table 2). Meanwhile, the non-polar contributions had a lower correlation with the binding free energy. Therefore, investigating the polar components in neuraminidase–OST complexes could provide further insights.

**Table 2:** Comparison of the coefficient of determination for polar and non-polar components^a^

Variants	IC_50_ change (fold)	Relative energy to the wild-type (kcal mol^−1^)					
		*E*_vdW_	*E*_surf_	*E*_NP_	*E*_Elec_	*E*_GB_	*E*_P_
G147R	0.880	−0.614	−0.042	−0.655	−3.667	4.693	1.027
H274Y	4.204	−1.138	0.063	−1.076	7.512	−4.523	2.989
G147R/H274Y	4.844	−1.928	0.093	−1.835	7.175	−3.522	3.652
***R***		−0.940	0.903	−0.928	0.902	−0.788	
***R*** ^**2**^		0.884	0.815	0.861	0.814	0.621	**0.987**

a *E*_vdW_ = van der Waals energy, *E*_surf_ = nonelectrostatic solvation energy, *E*_NP_ = non-polar energy contribution, *E*_ELEC_ = electrostatic energy, *E*_GB_ = electrostatic solvation energy, *E*_P_ = polar energy contribution.

Hydrogen bonds play a significant role in polar contributions to the binding free energy. We employed conservative criteria for hydrogen bonding, allowing a maximum distance of 3.0 Å and a minimum angle of 140°. Using this set up, we expected to reduce the noise/artefact of weak insignificant hydrogen bonds.

**Fig. 5:**
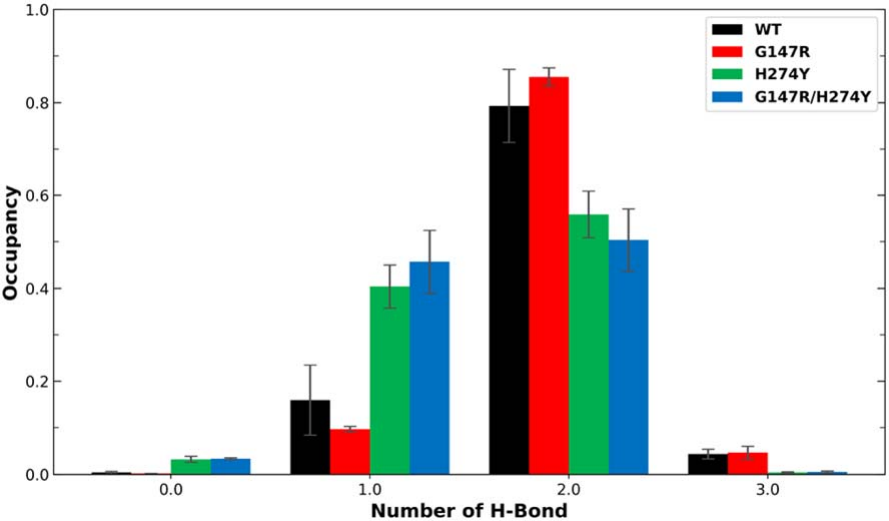
Number of H-bonds and their occupancy formed salt-bridge interactions in R224–E276. The error bars indicate the standard error means of the replicates.

Earlier studies have indicated that the mutation at position H274 to a larger residue disrupts the salt bridge formed by E276 and R224.^42^ Another study suggested that this salt bridge has minimal impact on the binding affinity of OST.^44^ We found the former phenomenon in our observation, as shown in Fig. 5. The WT and G147R preserved their interaction in salt-bridge formation with a high two hydrogen bonds. They even managed to form three hydrogen bonds, albeit not for a long time (less than 10% occupancy).

Meanwhile, H274Y showed a significant decrease in the formation of two hydrogen bonds. It also still maintained its one hydrogen bond longer than the WT for more than 40% occupancy, hence the salt bridge was not entirely disrupted. G147R/H274Y showed a greater decrease of its two hydrogen bonds than H274Y. Furthermore, its one hydrogen bond increased, substituting the loss interaction.

**Fig. 6:**
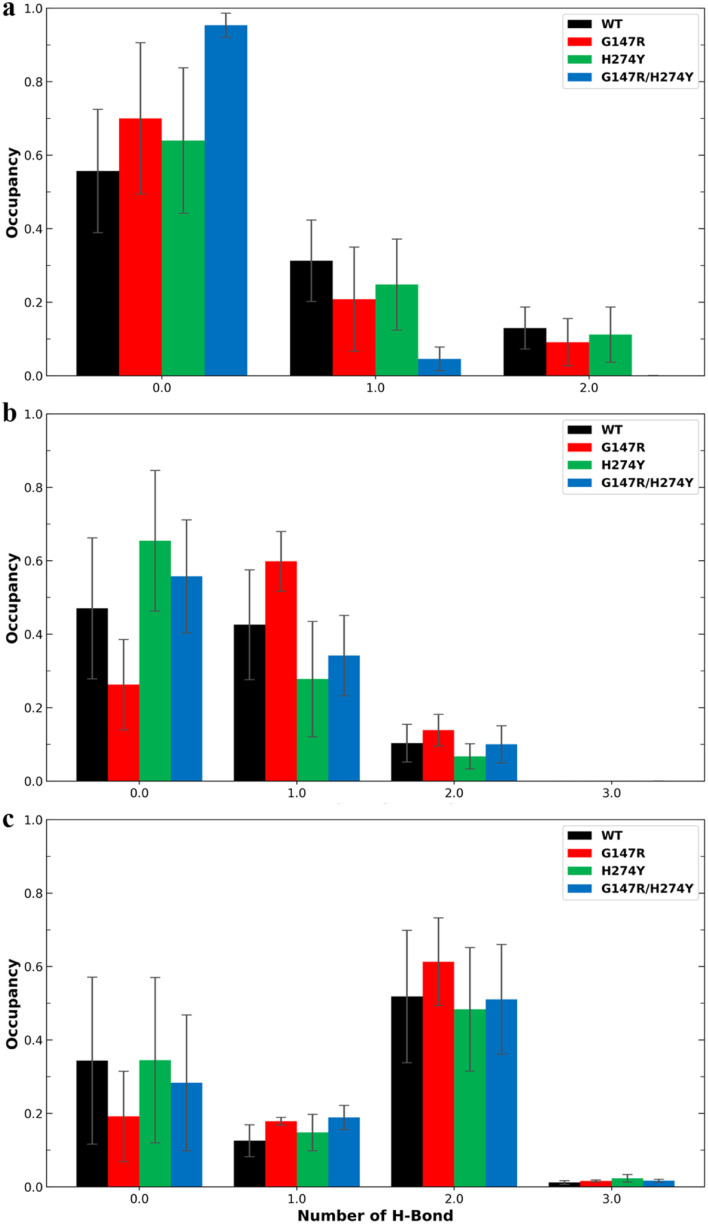
Number of hydrogen bonds and their occupancy formed by OST and R118 (a), R292 (b), and R371 (c). The error bars indicate the standard error means of the replicates.

Residue R118 is crucial for forming hydrogen bonds with the carboxyl moiety of OST. Together with residues R371 and R292, R118 interacts with the carboxyl group of sialic acid, making these residues pivotal in neuraminidase activity.^49^ In residue R118, the mutants generally showed lower hydrogen bond occupancy compared to the WT variant (Fig. 6a). The complementary effect of double mutation observed in the energy decomposition analysis of G147R/H274Y did reflect the hydrogen bond analysis. It showed that G147R/H274Y had less hydrogen bond interaction with R292 than the WT but more than H274Y (Fig. 6b). It was suspected that the conformational change observed in the cluster analysis (Fig. 4d) might affect this particular interaction. A similar result was also found on R371, where the hydrogen bond interaction reflected the energy decomposition analysis (Fig. 6c). Thus, the hydrogen bonds of these triad residues play a critical role in OST binding.

In the previous energy decomposition analysis, E119, R152 and K432 of G147R/H274Y showed a similar profile to G147R. Obviously, K432 did not have hydrogen bond interaction because it did not have an electronegative atom, except for its backbone. The hydrogen bond analysis also confirmed that no hydrogen bond interaction was observed among all the variants. For E119 and R152, the hydrogen bond was not quite similar to the energy decomposition (Fig. S1a and b†). Only in G147R did the hydrogen bond interaction in R152 align with the decomposition analysis. G147R/H274Y showed a reduction of interaction in E119, while in R152 it had comparable occupancy with the WT. Therefore, the interaction with E119 and R152 might not be dependent on hydrogen bonds.

As for E227, the energy decomposition analysis showed that all the mutants showed a decrease in interaction with OST, while E277 showed a strengthening interaction in contrast with the other mutants (Fig. S1c†). However, the hydrogen bond analysis did not align with these observations; whereby all the mutants showed a decrease in hydrogen bond interaction, even for G147R/H274Y in E277 (Fig. S1d†). The decrease in interaction was particularly disproportionate in the G147R mutant. Hence, for these residues, the hydrogen bond interaction might not be essential in OST binding, rather the electrostatic interaction was likely dominant.

We also analysed the distance between the OST and residues that did not show a correlation of the hydrogen bond interaction with the energy decomposition analysis. We suspected that the electrostatic interaction might play a dominant role in these residues. We set *C*-carboxyl and *C*-guanidine for the amino acid as the centre of the mass for the distance calculations. As for the OST moiety, we set N of the amino moiety and O in amide moiety as the centre of the mass. We calculated their distance probability and constructed a histogram chart to observe their distance propensity to these residues. The results were not very conclusive (Fig. S2†). All four residues did not show similar patterns with the interaction profile from the energy decomposition analysis. It may be that the interaction was more complicated than a simple term determined by distance.

**Fig. 7:**
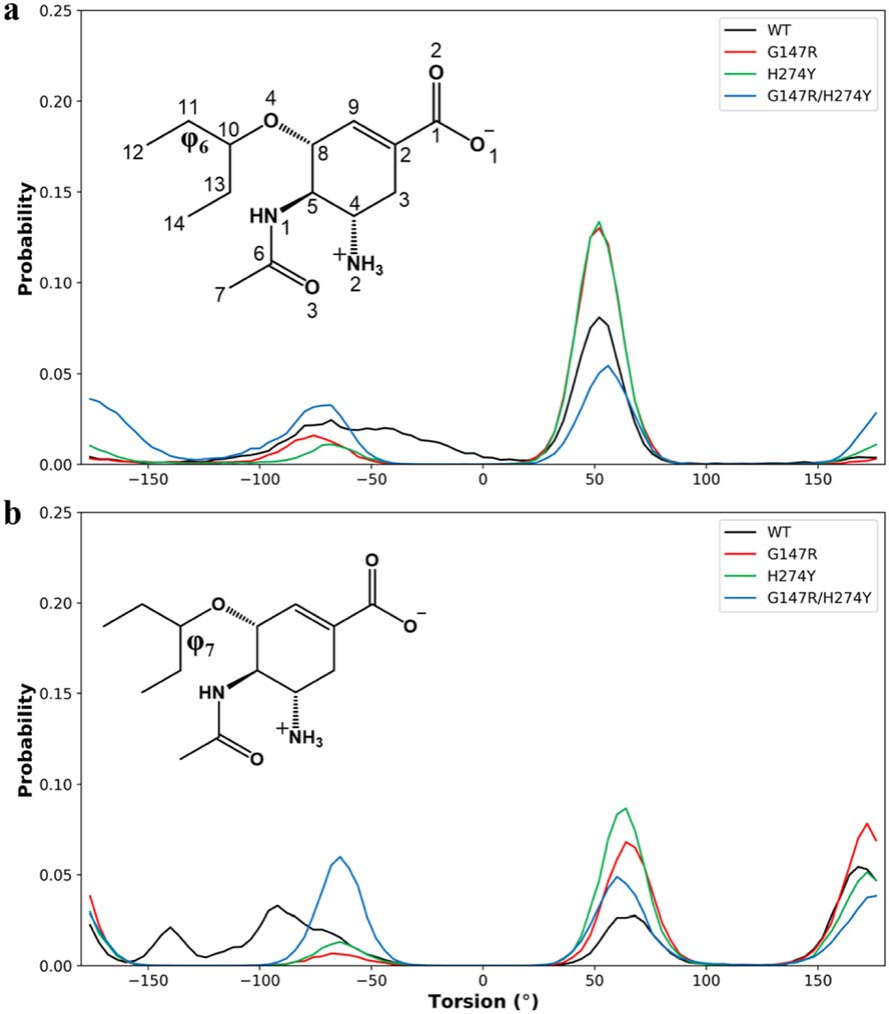
Normalized distribution of the dihedral angle of the pentyl moiety. The *ϕ*_6_ was formed by O4–C10–C11–C12 (a) and the *ϕ*_7_ was formed by O4–C10–C13–C14 (b).

We also looked for the dihedral angle of the pentyl moiety of OST as per a previous study.^44^ Park *et al.* (2009) suggested that the pentyl moiety of OST can suffer instability due to the infiltration of water into the binding site. Here we found that the dihedral angle of the pentyl moiety across all variants was relatively the same (Fig. 7). In *ϕ*_6_ (O4–C10–C11–C12), they all spread across different angles, with the highest peak at 50° (Fig. 7a). A slight decrease in the peak at 50° was observed for the WT and H274Y, albeit insignificant. The *ϕ*_7_ (O4–C10–C13–C14) also showed the same with a more distributed probability (Fig. 7b). From these observations, it can be indicated that the pentyl moiety of OST of all the variants was very flexible. There was no significant distinction between them. However, it showed a significant change among the variants. The amide moiety of OST also showed similar results. They all occupied a similar angle between the variants around 120° and 0° for *ϕ*_2_ (C8–C5–N1–C6) and *ϕ*_3_ (C5–N1–C6–O3) respectively (Fig. S3a and b†). This particular difference might be due to the length of our simulation (100 ns compared to 10 ns).

Noticeable changes were observed at the ether moiety connecting the pentyl and cyclohexene moieties. The *ϕ*_4_ (C5–C8–O4–C10) connecting cyclohexene showed a broadening peak in H274Y and G147R/H274Y, while the WT and G147R consolidated its peak around 100° (Fig. S3C†). Furthermore, *ϕ*_5_ (C8–O4–C10–C13) showed that the WT distributed its angle around −90° and 70°. Meanwhile, G147R and H274Y consolidated their peaks at 70° and G147R/H274Y distributed their peaks at 70° and 150° (Fig. S3d†). These findings indicated that despite the pentyl moiety being generally flexible, H274y and G147R/H274Y suffered more flexibility in the ether moiety, possibly due to the disturbance of the salt-bridge interaction.

A study by Park *et al.*^44^ indicated that the orientation of the pentoxyl moiety in the H274Y variant is unstable, characterized by the presence of three peaks. Furthermore, they found that water infiltration around the pentoxyl moiety of OST contributes to a decrease in OST affinity.^44^ Prior analysis suggested that this particular behaviour was not present in our study, rather the pentoxyl moiety showed a distributed dihedral angle across all the variants. We also analysed the number of water molecules within 3.4 Å of residue E276, assuming that close water infiltration near this residue could affect the binding free energy. Our results aligned with the previous study that the mutation in H274Y allowed the infiltration of water near E276. It also supported that the mutation in G147R alone did not confer this behaviour (Fig. S4†). However, we still suggest that water infiltration was neither the cause nor did it affect the binding affinity. Instead, the disruption of the R224–E276 salt bridge allowed water infiltration, which subsequently destabilized the pentoxyl moiety of OST.

In summary, the changes observed mainly occurred in the polar residue of neuraminidase. This was obvious since the residue content of the neuraminidase active side was predominantly polar residues, such as arginine, glutamate, asparagine. Hence, modifying the inhibitor to utilize these characteristics would be beneficial in combatting this variant. However, such modification obviously affects the pharmacokinetics and pharmacodynamics. These changes should be considered in drug development, especially in targeting neuraminidase.

## Conclusion

Molecular dynamics simulations combined with MM/PBSA binding free energy calculations successfully predicted binding free energies with a high correlation to experimental data. This correlation suggests that the generated analyses closely approximated the empirical study. Energy decomposition analysis indicated that the most influential interactions on OST resistance in mutant variants aligned with previous research, primarily involving polar interactions. We suspect that resistance in the G147R/H274Y variant is likely due to the disruption of the salt bridge of R224–E276, as prior hypothesized, that destabilized the pentoxyl moiety of OST. Moreover, this was also exacerbated by the significant decrease in interaction between OST and R118, which plays a crucial role in OST binding.

## Data availability

The data supporting this article have been included as part of the ESI.†

## Author contributions

Ardiana Ilham Nurrohman: investigation, formal analysis, visualization, writing – original draft, data curation. Hery Suwito: validation, writing – review & editing. Ni Nyoman Tri Puspaningsih: validation, writing – review & editing. Kautsar Ul Haq: conceptualization, methodology, investigation, formal analysis, supervision, project administration.

## Conflicts of interest

The authors declare that they have no known competing financial interests or personal relationships that could have appeared to influence the work reported in this paper.

## Supplementary Material

RA-014-D4RA07713J-s001
