# Supplementary material for: Molecular dynamics study on the effect of the N1 neuraminidase double mutant G147R/H274Y on oseltamivir sensitivity
Source: RSC Adv. 2024 Dec 10;14(52):39017–26. doi: 10.1039/d4ra07713j (PMC11629752; doi:10.1039/d4ra07713j)
Supplement: RA-014-D4RA07713J-s001 [file RA-014-D4RA07713J-s001.pdf]

Supplementary Information (SI) for RSC Advances.
This journal is © The Royal Society of Chemistry 2024

**Supplementary Materials**

# Molecular Dynamic Study on The Effect of N1 Neuraminidase Double Mutant G147R/H275Y to Oseltamivir Susceptibility

Ardiana Ilham Nurrohman[a,b], Hery Suwito[c], Ni Nyoman Tri Puspaningsih[b,c], Kautsar Ul Haq[a,c]*

[a.] Bioinformatic Research Group, University-CoE-Research Center for Bio-Molecule Engineering (BIOME), Universitas Airlangga 60115, Surabaya, Indonesia.

[b.] Proteomic Laboratory, University-CoE-Research Center for Bio-Molecule Engineering (BIOME), Universitas Airlangga, Surabaya 60115, Indonesia.

[c.] Department of Chemistry, Faculty of Science and Technology, Universitas Airlangga, Surabaya 60115, Indonesia.

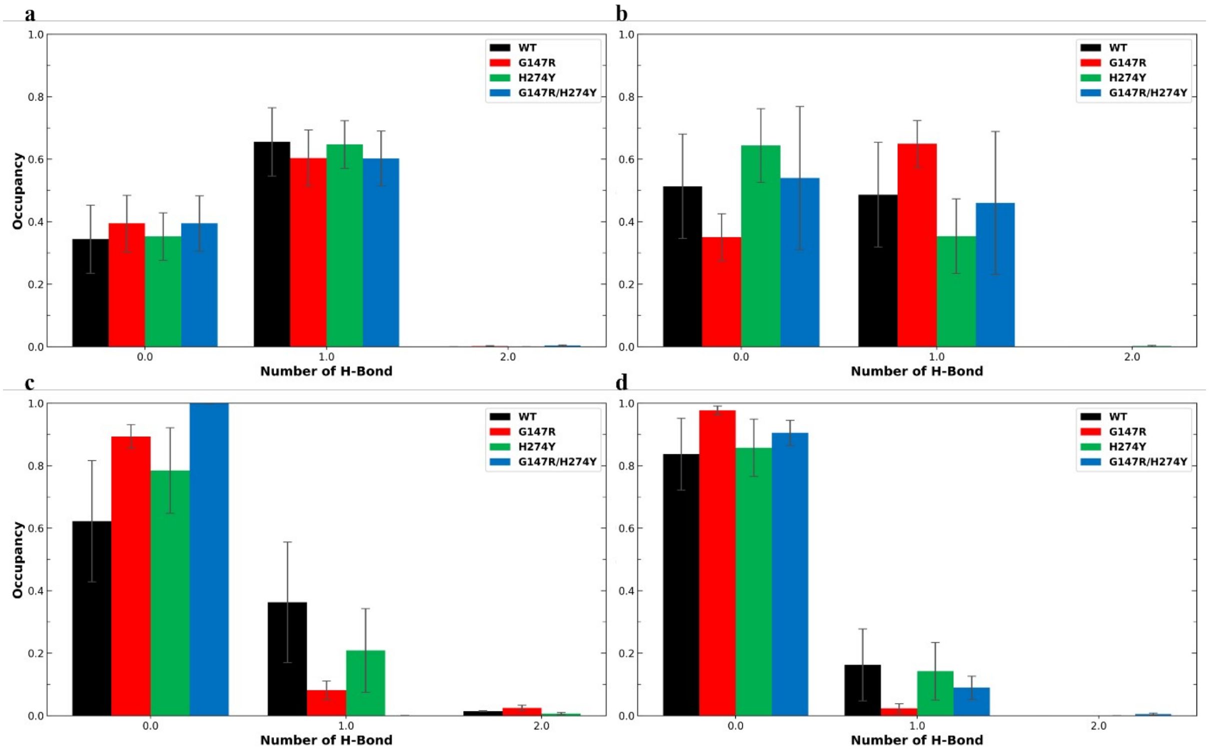


Figure S1. Hydrogen bonds occupancy formed by E119 (a), R152 (b), E227 (c), and E277 (d). The error bars indicated the standard error mean of replicates.

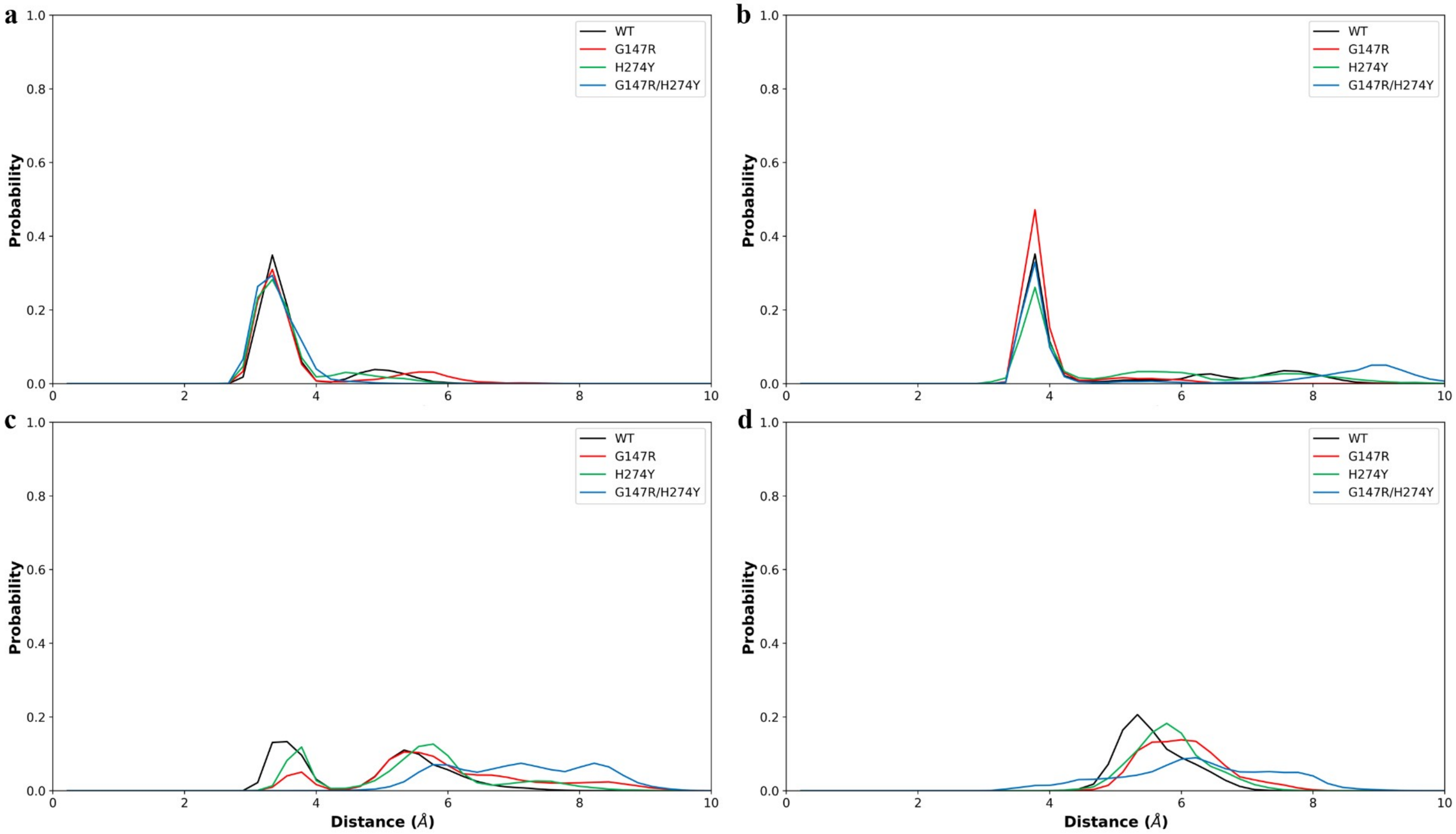


Figure S2. Normalized probability of the distance between N2 atom of OST with C-carboxyl of E119 (a), O3 atom of OST with C-guanidine of R152 (b), N2 atom of OST with C-carboxyl of E227 (c), and N2 atom of OST with C-carboxyl of E277 (d).

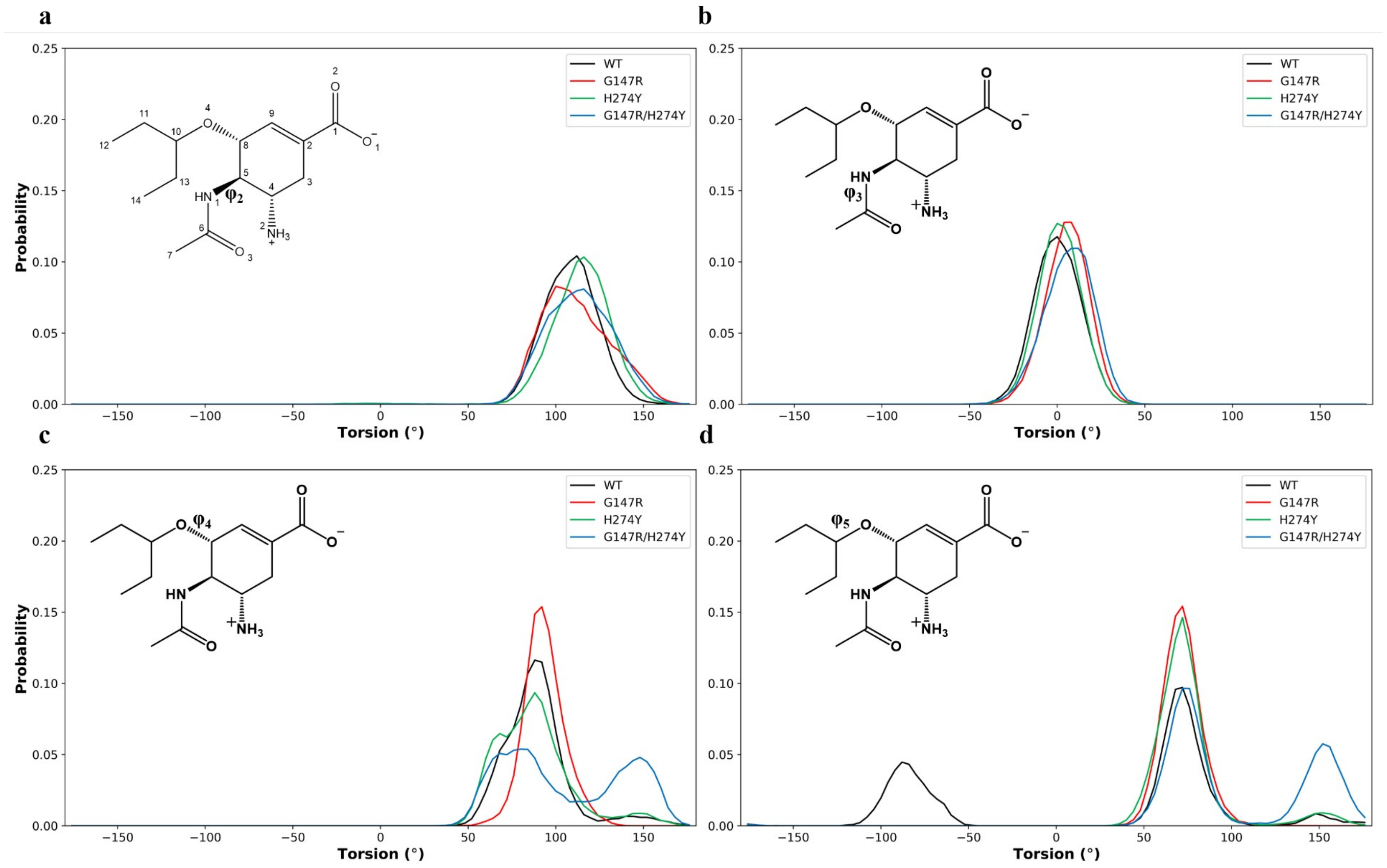


Figure S3. Normalized probability of the dihedral angle of $\varphi_4$ connecting pentoxyl moiety to cyclohexene (a), $\varphi_5$ connecting pentyl moiety to ether (b), $\varphi_2$ connection amide moiety to cyclohexene (c), $\varphi_3$ connecting acetyl moiety to amine moiety (d).

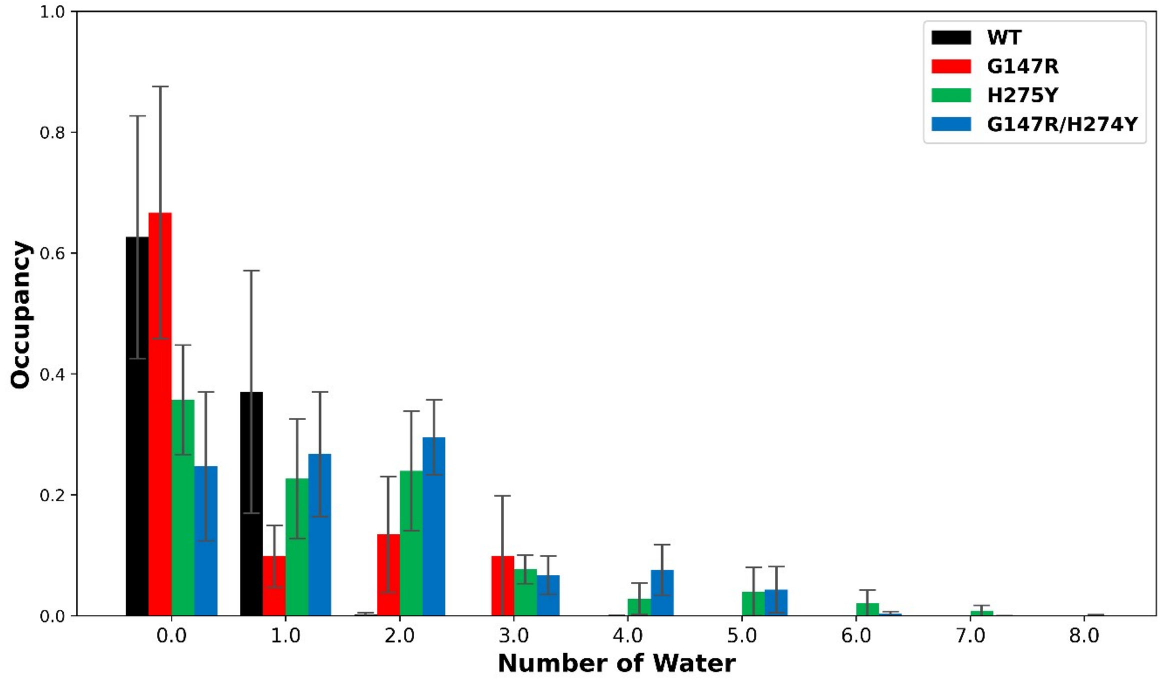


Figure S4. Normalized probability of water found near E276. Error bars indicated standard error mean of replicates.
